# Estimating the burden of lung cancer in Canada attributed to occupational radon exposure using a novel exposure assessment method

**DOI:** 10.1007/s00420-020-01537-2

**Published:** 2020-03-30

**Authors:** C. B. Ge, J. Kim, F. Labrèche, E. Heer, C. Song, V. H. Arrandale, M. Pahwa, C. E. Peters, P. A. Demers

**Affiliations:** 1 Institute for Risk Assessment Sciences (IRAS), Utrecht University, Yalelaan 2, 3584 CM Utrecht, Netherlands; 2 CAREX Canada, Simon Fraser University, Burnaby, Canada; 3 Occupational Cancer Research Centre, Cancer Care Ontario, Toronto, Canada; 4 Department of Epidemiology, Biostatistics and Occupational Health, McGill University, Montréal, Canada; 5 Institut de Recherche Robert-Sauvé en santé Et en sécurité du Travail, Montréal, Canada; 6 Department of Environmental and Occupational Health, School of Public Health, Université de Montréal, Montréal, Canada; 7 Department of Cancer Epidemiology and Prevention Research, Alberta Health Services, Calgary, Canada; 8 Dalla Lana School of Public Health, University of Toronto, Toronto, Canada; 9 Cumming School of Medicine, University of Calgary, Calgary, Canada

**Keywords:** Lung cancer, Radon, Occupational exposure, Disease burden

## Abstract

**Objective:**

Exposure to radon causes lung cancer. The scope and impact of exposure among Canadian workers have not been assessed. Our study estimated occupational radon exposure in Canada and its associated lung cancer burden.

**Methods:**

Exposed workers were identified among the working population during the risk exposure period (1961–2001) using data from the Canadian Census and Labour Force Survey. Exposure levels were assigned based on 12,865 workplace radon measurements for indoor workers and assumed to be 1800 mg/m^3^ for underground workers. Lung cancer risks were calculated using the Biological Effects of Ionizing Radiation (BEIR) VI exposure-age-concentration model. Population attributable fractions were calculated with Levin’s equation and applied to 2011 Canadian lung cancer statistics.

**Results:**

Approximately 15.5 million Canadian workers were exposed to radon during the risk exposure period. 79% of exposed workers were exposed to radon levels < 50 Bq/m^3^ and 4.8% were exposed to levels > 150 Bq/m^3^. We estimated that 0.8% of lung cancers in Canada were attributable to occupational radon exposure, corresponding to approximately 188 incident lung cancers in 2011.

**Conclusions:**

The lung cancer burden associated with occupational radon exposure in Canada is small, with the greatest burden occurring among those exposed to low levels of radon.

## Introduction

Radon is a radioactive gas that is naturally created by the decay of uranium in rocks and soil (National Toxicology Program 2015). Inhalation of radon and its radioactive decay progenies (hereafter: radon) damages the lung tissue and can lead to malignancy (Agency for Toxic Substances and Disease Registry (ATSDR) 2012). In outdoor air, radon is diluted in combination with other gases in the atmosphere but, when occurring indoors, it can accumulate to higher levels. Radon is heavier than air, so levels of radon in confined or underground spaces are often elevated compared to outdoor air (Bissett and McLaughlin 2010). Studies of underground miners in the 1920s revealed that exposure to radon as the first known cause of lung cancer and, in 1988, the International Agency for Research on Cancer (IARC) classified radon as a Group 1 carcinogen (carcinogenic to humans) with an established link to lung cancer (IARC Working Group on the Evaluation of Carcinogenic Risks to Humans 2009). After cigarette smoking, radon exposure is generally considered the most important risk factor for lung cancer and is the leading cause of lung cancer among never-smokers (Torres-Duran et al. 2014; World Health Organization 2009).

Residential radon exposure has been relatively well-characterized in Canada (Al-Arydah 2017; Gogna et al. 2019; Stanley et al. 2017), but occupational exposure levels, as well as the burden of cancer due to occupational exposure, is not as well understood in the Canadian context (Chen and Ford 2017). Reports of excess lung cancer mortality among uranium miners in the Northwest Territories during the 1980s and 1990 (Howe et al. 1986, 1987; Howe and Stager 1996) helped to prompt regulation of radon in high-exposure workplaces, such as mines. Radon exposure and associated lung cancer risk among workers in indoor workplaces have received considerably less attention, even though levels in these occupations can be sufficiently high to increase lung cancer risk (Marsh et al. 2017).

Previously reported estimates of the lung cancer burden associated with occupational radon exposure are typically calculated as a proportion of residential radon attributable lung cancers. For instance, Steenland et al. (2003) estimated that 2000 lung cancers annually (1.3%) in the United States are attributable to occupational radon exposure, based on a relative lifetime dose and, therefore, a burden of 20% compared to residential radon exposure (Samet 1989). More recently, Brown et al. (Brown et al. 2012b) estimated that 0.28–0.84% (0.56%, on average) of lung cancer deaths in the United Kingdom are caused by occupational radon exposure. Again, these figures were calculated as a proportion of the total annual radon-related lung cancer deaths (NRPB 2000). There is a need to estimate cancer burden attributed to occupational radon exposure in other jurisdictions using more detailed exposure estimates, thereby addressing important gaps in both the disease burden and exposure science literature.

The objective of the present study is to estimate the percentage of lung cancers in Canada attributed to occupational radon exposure using a novel approach to assess radon exposure for workers in underground and indoor workplaces, in addition to the traditionally highly-exposed occupations. The research presented here is part of the Burden of Occupational Cancer in Canada Study (Labreche et al. 2019).

## Methods

### Overall approach

The overall method used to estimate the fraction of lung cancers attributed to occupational radon exposure was similar to the method used to estimate occupational cancer burden from diesel engine exhaust exposure and other exposures in the same study (Kim et al. 2018; Labreche et al. 2019). In brief, the number of incident lung cancer cases attributed to occupational radon exposure was calculated as the product of the Population Attributable Fraction (PAF) and the number of incident lung cancers in Canadians aged 25 years or older in 2011 (Statistics Canada). Levin’s Equation [PAF = PrE(RR−1)/(1 + PrE(RR−1))] was used to calculate the PAF, where PrE equals the proportion of workers ever exposed and RR is the relative risk of lung cancer associated with radon exposure derived from the Biological Effects of Ionizing Radiation (BEIR) VI exposure-age-concentration risk model (National Research Council (US) Committee on Health Risks of Exposure to Radon 1999). Results from CAREX Canada’s radon exposure assessment, along with historical data from the Canadian Census and Labour Force Survey were used to estimate the total number of exposed Canadian workers during the risk exposure period (REP), defined as 1961–2001 assuming a latency of 10–50 years prior to lung cancer diagnosis. For the PAFs, 95% confidence intervals were calculated using Monte Carlo simulation of the PrE and the RR, described in detail elsewhere (Kim et al. 2018; Labreche et al. 2019). Our work received an ethics exemption from the University of Toronto Research Ethics Board and was performed in accordance with the ethical standards as laid down in the 1964 Declaration of Helsinki and its later amendments.

### Exposure assessment

Radon exposure assessment was performed as part of the CAREX Canada project (Peters et al. 2015). Briefly, exposure was assessed on the population reported in the 2006 Canadian census, where information for the Canadian workforce was available by sex, province, industry, and occupation. Exposure to radon was estimated for Canadian workers in two distinct exposure scenarios: high-risk occupations where workers have the potential for high exposure (> 800 Bq/m^3^) in underground/confined workspaces, and low-risk occupations where workers are exposed to radon in indoor air at lower levels (> 0 to > 800 Bq/m^3^). For the high-risk occupations, two authors (CG, CP) identified occupations and industries with potential for high radon concentrations using information from the Canadian National Dose Registry (that contains doses experienced by radiation-exposed workers) (Health Canada 2019), published literature (Navaranjan et al. 2016), and government reports/grey literature (Beaugrand and Sutton 2012; Brown et al. 2012a; Canadian Broadcast Corporation 2014; Kalinowski 2014; U.S. Department of Health and Human Services 1987). They then estimated the proportion exposed in each detailed occupation and industry intersection.

For the estimation of the number of indoor workers exposed to radon, workers in indoor working environments where radon has the potential to accumulate were identified by occupation and industry by two authors (CP, PD). Distributions of radon concentrations were calculated, by province, with radon measurements from the Canadian Federal Building Survey, where 12,865 indoor federal workplaces had been monitored at the time of estimation (Health Canada 2014; Whyte et al. 2019). The data from this survey of federal buildings were used to model lognormal exposure distributions for workplace radon. From each province-specific radon exposure distribution, the proportion of workers exposed to specific ranges of radon were calculated (> 50–100, > 100–150, > 150–200, > 200–400, > 400–800, and > 800 Bq/m^3^). For subsequent calculation of lung cancer relative risks, the midpoints of these exposure ranges were assigned as the average radon concentrations for the exposure groups. For the unbounded category of > 800 Bq/m^3^, the province-specific mean of the radon measurements exceeding 800 Bq/m^3^ in the federal building survey was assigned. For high-risk workers, we approximated the average of the unbounded exposure category (> 800 Bq/m^3^) to be 9/4 times the value of the lower bound; therefore, the average radon exposure level estimated for these high-risk workers is 800 Bq/m^3^ × 9/4 = 1800 Bq/m^3^.

### Calculation of ever-exposed population

Methods for population modeling are described in detail elsewhere (Kim et al. 2018; Labreche et al. 2019). Briefly, historical workforce data were obtained from Canadian census data from 1961, 1971, 1981, 1991, and 2001. Workforce data for between-census years were linearly interpolated. To avoid double-counting workers, after 1961, only new hires were added to the working population model. New hire information was available from the Canadian Labour Force Survey by sex, industry, and age group (Statistics Canada 1976–2003). Annual workforce data in the REP (1961–2001) were merged, by industry and occupation, to aforementioned exposure assessment results to obtain the total number of workers ever exposed, by sex, province, industry, and occupation. The population was further adjusted for the probability of survival to the target year (2011), which was estimated using Canadian life tables (Statistics Canada Demography Division 1960–2002). The reference population was the population aged 25 years and older reported in the 2011 Canadian census, which included persons alive in 2011 who were ever of working age during the REP.

### Relative risk of lung cancer

The BEIR VI exposure-age concentration model (National Research Council (US) Committee on Health Risks of Exposure to Radon 1999) was used to derive relative risks of lung cancer for radon exposure. When the rate of exposure is less than 0.5 working levels (0.5 working levels = 85 working level months (WLM) = 5 millisieverts) as is the case in occupational exposure scenarios, the BEIR VI model may be represented as RR = 1 + 0.0768w*Ф, where w is the cumulative exposure (in WLM) weighted based on time-since exposure factors (5–14 years = 1; 15–24 years = 0.78; 25 + years = 0.51) and Ф is a factor accounting for age at the time of risk estimation (< 55 years = 1; 55–64 years = 0.57; 65–74 years = 0.29; 75 + years = 0.09). Conversion from 800 Bq/m^3^ to WLM was performed based on an assumption of 2000 h of work per year, leading to a correspondence of 1 Bq/m^3^ to 0.00126 WLM (ICRP 1993). Total cumulative exposure and time since last exposure information was calculated from average job durations, which were estimated by occupation, sex, and age group using data from the lifetime job histories of controls in the National Enhanced Cancer Surveillance System (Johnson 1998).

## Results

Approximately 26,000 high-risk workers and 15.5 million indoor workers were exposed to radon during the REP (Table 1). The majority of exposed indoor workers (79%) were in the lowest exposure group with an exposure level of > 0–50 Bq/m^3^. Very few exposed workers (0.5%) in indoor workplaces had exposures above 400 Bq/m^3^. The lung cancer attributable fraction and number of cases for indoor workers by Canadian province are shown in Table 2.

**Table 1:** Lung cancer cases attributable to occupational radon exposure by exposure level. Results presented as both number of cases and attributable fraction of cases

	Exposure level (Bq/m^3^)	NeREP (% of total exposed)^a^	Attributable lung cancers^a^ (% of total cases)	Population attributable fraction (%)
Indoor workers	> 0–50	12,284,327 (79%)	74 (39%)	0.31
	> 50–100	1,979,557 (13%)	36 (19%)	0.15
	> 100–150	604,486 (4%)	18 (10%)	0.08
	> 150–200	254,953 (2%)	11 (6%)	0.05
	> 200–400	272,887 (2%)	20 (10%)	0.08
	> 400–800	65,678 (0.4%)	9 (5%)	0.04
	> 800	14,109 (0.2%)	5 (3%)	0.02
High-risk workers	> 800	26,109 (0.2%)	16 (9%)	0.07
	Total	15,502,892 (100%)	188 (100%)	0.80

^a^Number of cases may not add up to total because of rounding

**Table 2:** Number and fraction of cancers attributable to occupational radon exposure for indoor workers by the included Canadian province

Province	NeREP (% of total exposed)^a^	Attributable lung cancers^a^ (% of total cases)	Attributable fraction (% of total cases in region)
Alberta	1,639,001 (10.6%)	18 (10.6%)	0.99
British Columbia	1,766,097 (11.4%)	14 (8.4%)	0.52
Manitoba	608,106 (3.9%)	15 (8.6%)	1.73
New Brunswick	344,117 (2.2%)	4 (2.3%)	0.61
Newfoundland	236,514 (1.5%)	3 (1.6%)	0.71
Nova Scotia	459,550 (3.0%)	5 (2.8%)	0.54
Ontario	6,117,923 (39.5%)	48 (28.2%)	0.60
Prince Edward Island	57,922 (0.4%)	1 (0.3%)	0.38
Quebec	3,744,181 (24.2%)	49 (28.4%)	0.68
Saskatchewan	503,372 (3.3%)	15 (8.7%)	1.94
Total	15,476,783 (100%)	172 (100%)	0.73

^a^Number of cases may not add up to total because of rounding

Average lung cancer relative risk for the high-risk workers was 1.60. Average cancer risks for the indoor workers were 1.01 and 1.42 for the lowest and highest exposed groups, respectively.

The fraction of lung cancers attributable to radon exposure among high-risk workers was 0.07% (95%CI = 0.05–0.25%), which equated to approximately 16 new cancer cases in 2011. These cancers occurred almost completely (98%) in underground mining and oil and gas extraction industries. The remaining percentages of lung cancers occurred in workers from transportation/warehousing industries (1.2%) and utilities (0.3%). The total attributable fraction for lung cancer for all radon exposed indoor workers was 0.73% (95%CI = 0.59–2.12%), which translated to 172 new lung cancer cases in 2011. The majority of attributable cases (110 of 172) were estimated to be due to indoor exposures to concentrations at or lower than 100 Bq/m^3^ (Table 1).

## Discussion

Combining high risk and indoor working populations, our results showed that approximately 15.5 million Canadian workers were exposed to radon at some point from 1961 to 2001 and 188 cases of lung cancer in 2011 were attributable to this exposure. Our total attributable fraction estimate (0.80%) is within the range of those reported from other studies in the United Kingdom (0.56%) and the United States (1.2%) (Brown et al. 2012b; Steenland et al. 2003). When compared to previous studies, the present analysis used improved methods to estimate the occupational contribution to radon-induced lung cancers. The majority of indoor workers were exposed to relatively low levels of radon in the workplace.

Our results show that exposure above 200 Bq/m^3^, the current Health Canada guideline for intervention, is estimated to account for 20% of the attributable lung cancer cases associated with indoor exposures. If the limit is further lowered to 100 Bq/m^3^, 37% of the cases could be prevented. Our estimates are similar with those from the UK Burden Study, where the authors noted that around 10% of all radon-attributable lung cancers are avoidable because most attributable cases were associated with radon exposures below 200 Bq/m^3^ (Brown et al. 2012a). An analysis of residential radon exposure in Canada found that remediating homes above the Health Canada guideline of 200 Bq/m^3^ down to background levels (10–30 Bq/m^3^) would prevent 28% of radon-attributable lung cancer deaths (Peterson et al. 2013). Slightly over half (52%) of the radon-attributable lung cancer deaths would require remediation of all homes above 50 Bq/m^3^ (Chen et al. 2012). Radon exposure awareness and building remediation initiatives in regions with high geologic potential for radon emission may have the benefit of reducing both the occupational and residential burden of radon-related lung cancer.

Our study included detailed and quantitative radon exposure estimates for Canadian workers by job, industry, and geographical region. Exposures for indoor workers, where over 90% of radon-attributable cases occurred, were modeled with more than 12,000 workplace radon measurements collected in workplaces across Canada. Various registries, including the Canadian national census, Labour Force Survey, and National Enhanced Cancer Surveillance System were also linked to fully characterize the Canadian workforce in detail. There are, however, several limitations in our work. Data collection for the workplace radon measurements were carried out year-round in air-conditioned buildings and in the fall/winter in buildings with natural ventilation. However, building ventilation information was not available in our dataset and we were unable to calculate the ratio of the two building types or perform sensitivity analysis using radon measurements from air-conditioned buildings only. The fall/winter measurements may overestimate average annual radon concentrations in buildings with natural ventilation, leading to overestimation of attributable risk in indoor workers. However, a recent study based on Canadian residential radon measurements suggested that no seasonal adjustments are needed for radon concentrations in population-level studies because radon levels were not found to be consistently higher in the majority of homes in winter (Stanley et al. 2019). We were also unable to account for historical changes in radon exposure. There is evidence that newer Canadian homes tended to have higher radon concentration compared to older homes (Stanley et al. 2019). If the same applies to workplace buildings, then past exposure may have been overestimated in our study, leading to an overestimation of attributable risk and cases. For high-risk underground workers, assuming a constant exposure likely underestimated past exposures and, therefore, attributable cancer cases.

In addition to being the leading cause of lung cancer, cigarette smoking also acts synergistically with radon exposure in causing lung cancer (Leuraud et al. 2011; National Research Council (US) Committee on Health Risks of Exposure to Radon 1999). However, smoking data was not available for our population. The BEIR VI standard exposure-age-concentration model also did not account for smoking status. In combining the BEIR VI model-derived risks with overall rates of lung cancer among both smokers and non-smokers for our attribute risk calculations, we assumed a constant ratio of excess risk for smokers and non-smokers, which is consistent with a multiplicative model interaction between radon exposure and smoking (National Research Council (US) Committee on Health Risks of Exposure to Radon 1999). There are, however, suggestions that the interaction between smoking and radon may be super-additive but sub-multiplicative (Leuraud et al. 2011; National Research Council (US) Committee on Health Risks of Exposure to Radon 1999). If this were true, our results would overestimate the risk for smokers and underestimate the risk for non-smokers, leading to an overestimate of overall risk because radon-related lung cancer burden is much higher in smokers than in non-smokers (Mendez et al. 2011; National Research Council (US) Committee on Health Risks of Exposure to Radon 1999).

In summary, our work showed a small lung cancer burden associated with occupational radon exposure and most of the burden occurred among workers exposed at relatively low levels of radon in indoor workplaces. A combination of smoking cessation strategies with identification and remediation of workplaces with high radon concentrations, particularly in regions with high underlying geological potential for radon emission, may help reduce this burden.
